# Impaired toll like receptor-7 and 9 induced immune activation in chronic spinal cord injured patients contributes to immune dysfunction

**DOI:** 10.1371/journal.pone.0171003

**Published:** 2017-02-07

**Authors:** Gozde Gucluler, Emre Adiguzel, Bilgi Gungor, Tamer Kahraman, Mayda Gursel, Bilge Yilmaz, Ihsan Gursel

**Affiliations:** 1 THORLAB, Department of Molecular Biology and Genetics, Bilkent University, Ankara, Turkey; 2 Gaziler Physical Medicine and Rehabilitation Education and Research Hospital, Ankara, Turkey; 3 Department of Molecular Biology and Genetics, Middle East Technical University, Ankara, Turkey; 4 University of Health Sciences, Gulhane Medical School, Department of Physical Medicine and Rehabilitation, Gaziler Physical Medicine and Rehabilitation Education and Research Hospital, Ankara, Turkey; University of Toronto, CANADA

## Abstract

Reduced immune activation or immunosuppression is seen in patients withneurological diseases. Urinary and respiratory infections mainly manifested as septicemia and pneumonia are the most frequent complications following spinal cord injuries and they account for the majority of deaths. The underlying reason of these losses is believed to arise due to impaired immune responses to pathogens. Here, we hypothesized that susceptibility to infections of chronic spinal cord injured (SCI) patients might be due to impairment in recognition of pathogen associated molecular patterns and subsequently declining innate and adaptive immune responses that lead to immune dysfunction. We tested our hypothesis on healthy and chronic SCI patients with a level of injury above T-6. Donor PBMCs were isolated and stimulated with different toll like receptor ligands and T-cell inducers aiming to investigate whether chronic SCI patients display differential immune activation to multiple innate and adaptive immune cell stimulants. We demonstrate that SCI patients' B-cell and plasmacytoid dendritic cells retain their functionality in response to TLR7 and TLR9 ligand stimulation as they secreted similar levels of IL6 and IFNα. The immune dysfunction is not probably due to impaired T-cell function, since neither CD4^+^ T-cell dependent IFNγ producing cell number nor IL10 producing regulatory T-cells resulted different outcomes in response to PMA-Ionomycin and PHA-LPS stimulation, respectively. We showed that TLR7 dependent IFNγ and IP10 levels and TLR9 mediated APC function reduced substantially in SCI patients compared to healthy subjects. More importantly, IP10 producing monocytes were significantly fewer compared to healthy subjects in response to TLR7 and TLR9 stimulation of SCI PBMCs. When taken together this work implicated that these defects could contribute to persistent complications due to increased susceptibility to infections of chronic SCI patients.

## Introduction

Infectious complications, predominantly septicemia and pneumonia are the most common causes of death in the years following spinal cord injury (SCI) [1,2]. Urinary and respiratory infections are the most abundant problems of re-hospitalizations of these patients. Therefore, it is likely that there is an increased susceptibility to infections in patients with chronic SCI due to impaired sensing of pathogen associated molecular patterns (PAMPs) of the microbial world [3].

This increased susceptibility to infections following SCI has prompted the researchers to consider the reasons leading to reduced immune response in this patient group. Indeed, studies revealed that there are several abnormalities in immune system that might increase the rate and severity of infections in patients with SCI. Natural killer cell counts and cytotoxicity levels were found to be decreased [4], whereas there were contradictory reports about the relationship between the level of injury and immune dysfunction [5,6].

When the possible contribution of the level of injury to immune dysfunction is taken into account, possible effect of another important factor-autonomic nervous system dysfunction- has to be kept in mind. Although there was some effort to clarify this issue, it is still unresolved [7].

In most of the central nervous system diseases (i.e. multiple sclerosis, Alzheimer’s disease, stroke, and HIV encephalitis), notable activation of innate immunity occurs [8, 9]. In SCI, strikingly beside activation of innate immunity, an immune impairment is also present. Increased levels of circulating pro-inflammatory cytokines and autoantibodies have been reported which supports these immune changes [10, 11, 12]. Despite elevated inflammatory processes, patients with chronic SCI frequently show a state of immune suppression and a susceptibility to infections [13, 14].

Accordingly, we aimed to define the role of nucleic acid sensors triggering innate immune response from SCI PBMC in this cross-sectional study. We found that SCI patients with level of injury above T-6 displayed impaired TLR7 and TLR9 mediated immune activation.

## Materials and methods

### Patients and controls

Subjects were recruited from spinal cord injury rehabilitation unit of a national rehabilitation center and healthy controls were included from the same hospital employee. Patients inclusion criteria were as follows: 1) neurologically complete spinal cord injury (AIS A, as defined by the American Spinal Injury Association, Atlanta, GA, USA) occurring 6 or more months prior to the study [15], 2) level of injury above T-6, 3) 18 years of age or older. Exclusion criteria were fever in 7 days preceding study procedures; current pressure ulcers; having vaccine therapy in the last three months; use of substances that may influence the immune system such as corticosteroids, alcohol and chemotherapies in the last nine months. All patients have received corticosteroid treatment in acute injury for anti-edema purpose when they were first admitted to the hospital. To these patients, administration of methylprednisolone was done according to the NASCIS 2 protocol. All patients underwent a general examination and a full American Spinal Injury Association (ASIA) examination were conducted before they were included the study. Written informed consent was obtained from each participant and the study protocol was approved by Keçiören Training and Research Hospital Ethical Committee with the decision number 249 on 13.03.2013. Venous blood samples were drawn under non-fasting conditions between 7:00–8:00 am from all subjects. Laboratory personnel were blinded to the case-control identification.

### PBMC isolation

Blood samples were transferred to 15ml falcon tubes containing lymphocyte separation medium (Lonza, 17-829F) and immediately centrifuged at 1500 RPM for 30 minutes with the break off. At the end of the centrifuge, three layers were obtained. The lymphocyte fraction was gently transferred into new tubes and washed twice with fresh media (1500 RPM for 10 min). The supernatant was discarded and the cells were adjusted to 1x10^6^/ml before further analysis.

### Treatment of cells with stimulants

Isolated PBMCs were seeded (2x10^5^/well/200μl) in 96-well plate and incubated with i) R848 (5μg/ml, Enzo), ii) D-type CpG ODN (3μM, ID: D35 sequence, Alpha DNA, 5’ggTGCATCGATGCAGGGGgg-3’, lower letters represents phosphodiester linkages, capital letters phosphorothioate linkages, hereafter D35), iii) PHA (5μg/ml, Roche) and iv) LPS (1μg/ml, Sigma) for 24 hours for cytokine ELISA studies. The cell supernatants were analyzed for human IL6, IL10, IFNα, IFNγ and IP10 production.

For human IFNγ and IP10 intracellular cytokine studies by flow cytometry (BD Accuri C6 Flow Cytometry, Becton Dickenson), 10^6^/ml PBMCs were incubated with R848 (5μg/ml)or D35 (3μM) for 12 hours. Then, Brefeldin A (10μl/ml, Sigma) was added onto each sample and the cells were further incubated for eight hours.

To assess T-cell function via IFNγ intracellular staining in response to PMA-Ionomycin, 10^6^/ml PBMCs were incubated with PMA (10ng/ml, Sigma), Ionomycin (0,5μM, Sigma) and Brefeldin A (10μg/ml) for 6 hours in 1ml media. Then, the cells were collected for intracellular cytokine staining analysis by FACS.

### ELISA studies

2HB 96-well plates (ThermoFisher Scientific) were coated with Abs specific for human IL6, IL10, IFNα, IFNγ and IP10 for 4 h at RT or o/n at 4°C. In S1A Table, specificities and concentrations of each antibody used for ELISA analyses were listed. The plates were blocked with 1x PBS-5% BSA for 2h at RT, washed 5x with 1x PBS-0.025% Tween 20 and then cell supernatants were layered onto the wells, recombinant standard proteins (S1B Table) in duplicates were plated via serial dilutions (1/2x dilution, 11 series). Following incubation at 4°C o/n, plates were washed and biotin-labeled anti-cytokine Abs (S1A Table) were added and incubated for 2h at RT. Streptavidin-Alkaline Phosphatase (1:1000x diluted, ThermoFisher Scientific) was added after washing the plates. Following incubation 1h at RT and washing, ELISA plates were developed with the addition of Phosphatase substrate (1:5000x, ThermoFisher Scientific). Optical density values for samples and standard cytokines were detected on a Spectramax ELISA reader (Molecular Devices) at 405nm.

### Intracellular cytokine staining studies

For IFNγ and IP10 intracellular cytokine staining studies, cells were incubated with the respective ligands as indicated above. Following centrifugation, supernatants were discarded and cell pellets were resuspended in fixation medium A (100μl, ThermoFisher Scientific) and incubated in dark at RT for 15 mins. PBS-BSA-Sodium azide buffer (2 ml) was added into each tube and then washed twice. At the end of the second washing step, permeabilization medium B (50μl, ThermoFisher Scientific) containing anti-IFNγ-FITC and anti-CD4-PE cocktail were added onto cells. For IP10 staining, anti-CD14-FITC and anti-IP10-PE (S1A Table) were added and the samples were incubated in dark for 30 mins. Cells were washed twice, resuspended in 500μl PBS-BSA-Sodium azide buffer, transferred to FACS tubes and analyzed in BD Accuri 6 Flow Cytometry.

### CD83/HLA-DR studies

Healthy and SCI PBMCs (10^6^ /ml) were treated with R848 (5μg/ml) or D35 (3μM) in 1ml final volume of media for 24h. Cells were fixed and washed as described above and stained with anti-CD83-PE and anti-HLA-DR- FITC Ab. Cells were washed twice, resuspended in 500μl PBS-BSA-Sodium azide, and analyzed in BD Accuri 6 Flow Cytometry.

### Statistical analysis

Statistical analysis was performed using GraphPad Software (GraphPad Inc.). All continuous variables were presented as mean and standard deviation. Categorical data was presented using number and percent. Mann-Whitney U test was used for comparison of between group results and two-way ANOVA with Bonferroni’s correction was used for comparison of within-group results. Categorical variables were compared with the X2 test. p<0.05 was considered to be significant.

## Results

Seventeen patients with SCI and thirteen healthy controls were included in the study. Age and sex distributions of patients and healthy participants and etiologies of the injuries are shown in Table 1 and S1 Fig. There was no statistically significant difference in age and sex distribution between the SCI group and the healthy controls (Mann-Whitney U test, p = 0.16, Chi-square test, p = 0.98, respectively). All patients were complete (AIS A) spinal cord injured individuals. Complete blood counts of the SCI and healthy control group were shown in Table 2. There was no statistical significance between blood counts of the groups (p>0.05, Mann-Whitney U test).

**Table 1:** Baseline characteristics of the patients and control group.

	SCI Patients (n = 17)	Healthy Controls (n = 13)	p
Age (median, (min-max))	37.0 (21.0–45.0)	29.0 (24.0–49.0)	0,14*
Sex (%male; %female)	82.4; 17.6	53.8; 46.2	0,09**
Months from injury (median, (min-max))	72.0 (10.0–288.0)	NA	
Etiology (%)			
Motor vehicle collisions	47.0	NA	
Fall from an elevated height	29.4	NA	
Gunshot wound	11.8	NA	
Diving into shallow water	11.8	NA	

Abbreviation: NA, not applicable

* Mann-Whitney U test

**Chi-square test

**Table 2:** Hematological characteristics of the patients and the control group.

Blood variable	SCI patients	Healthy control	p
Haemoglobin (g/dl)	12.9±1.2	14.1±1.4	>0.05*
Erythrocytes (X10^6^/μL)	4.5±0.4	4.8±0.3	>0.05*
Trombocytes (X10^3^/μL)	255.8±43.7	269.8±79.5	>0.05*
Leukocytes (X10^3^/μL)	6.3±1.1	6.9±2.3	>0.05*
Leukocytes subsets (X10^3^/μL)			
Lymphocytes	2.4±0.6	2.4±0.9	>0.05*
Neutrophils	3.2±0.8	3.6±1.4	>0.05*
Eosinophils	0.2±0.1	0.2±0.1	>0.05*
Monocytes	0.5±0.1	0.5±0.1	>0.05*

* Mann-Whitney U test

**Fig 1:**
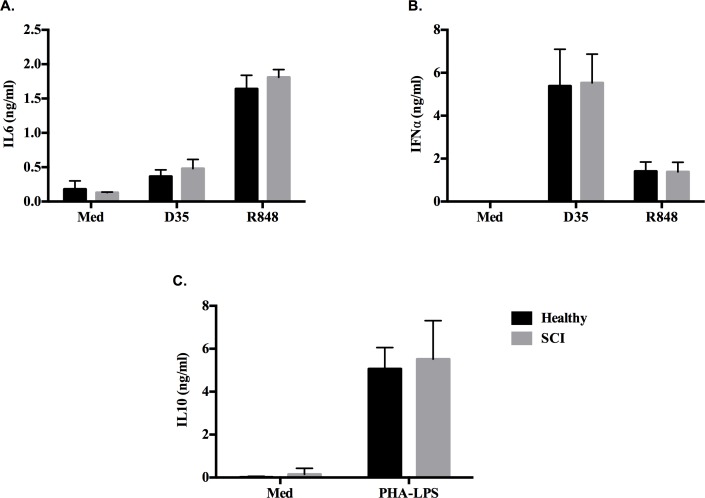
Healthy and SCI donor PBMCs respond similarly to different immune cell stimulants. PBMCs (2x10^5^/200μl/well) were incubated with different immune stimulants for 24h. (A) IL6 and (B) IFNα secretion in response to R848 (5μg/ml) and D35 (3μM), (**C)** IL10 production in response to PHA-LPS (5μg/ml-1μg/ml).

Cytokine ELISA results revealed that SCI patients`B cells and pDCs compared to healthy control cells had no significant difference in response to R848 (TLR7 ligand, 5μg/ml) and D35 (TLR9 ligand, 3μM) stimulation, since they induced similar levels of IL6 and IFNα (Fig 1A and 1B, respectively). Moreover, the ability of CD4^+^ T-cells to secrete immunomodulatory cytokine IL10 in response to PHA+LPS implicated that SCI patients does not have any Treg dependent dysregulation compared to healthy PBMCs (Fig 1C), indicating that the cell sources of suspected immune dysfunction is not mainly due to impaired B cell, pDC or altered CD4^+^ Treg activity (Fig 1).

**Fig 2:**
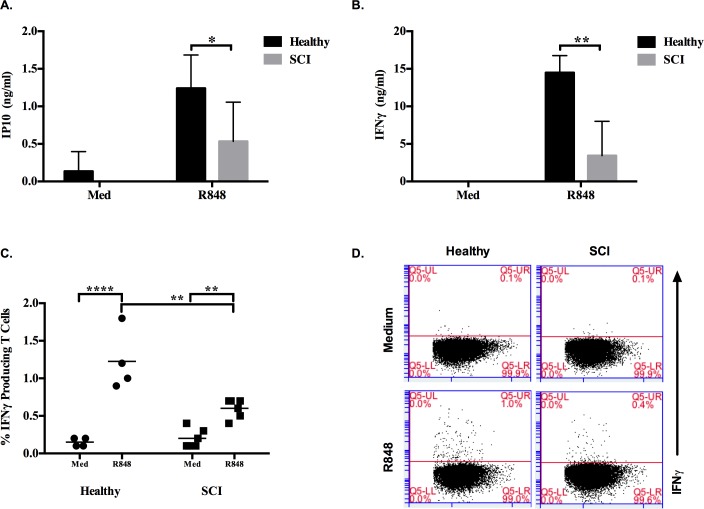
Healthy and SCI individuals show discrepant IFNγ production in response to TLR7 ligand stimulation. PBMCs (2x10^5^/200μl/well) were incubated with R848 (5μg/ml) for 24h. (A) IP10 and (B) IFNγ secretion levels as assessed by ELISA from healthy (n = 5) and SCI (n = 10) donors. PBMCs (10^6^/ml) were stimulated with R848 (5μg/ml) for 20h, at 12h Brefeldin A (10μl/ml) was added. (C) Intracellular IFNγ levels of healthy (n = 4) and SCI (n = 6) donors, (D) Representative dot plots demonstrating IFNγ+/CD4^+^ T cells. During analyses 50000 cells were acquired. Mann-Whitney test was used to identify significance between healthy and SCI responses to R848. Two-way ANOVA with Bonferroni Correction was used to test the significance of R848 responses compared to medium alone group (*, p≤0.05; **, p≤0.01; ****, p≤0.0001).

When IP10 secretion levels between healthy and SCI PBMCs in response to R848 were compared, there was 2.3 fold less IP10 production from SCI PBMCs (Fig 2A). Strikingly, secreted IFNγ was 4.2 fold less from SCI patients compared to healthy control groups (Fig 2B). Moreover, this downtrend was confirmed at cellular level by intracellular cytokine staining as healthy controls had significantly higher IFNγ producing T cells then SCI patients in response to TLR7 ligand stimulation (Fig 2C and 2D, S2 Fig).

**Fig 3:**
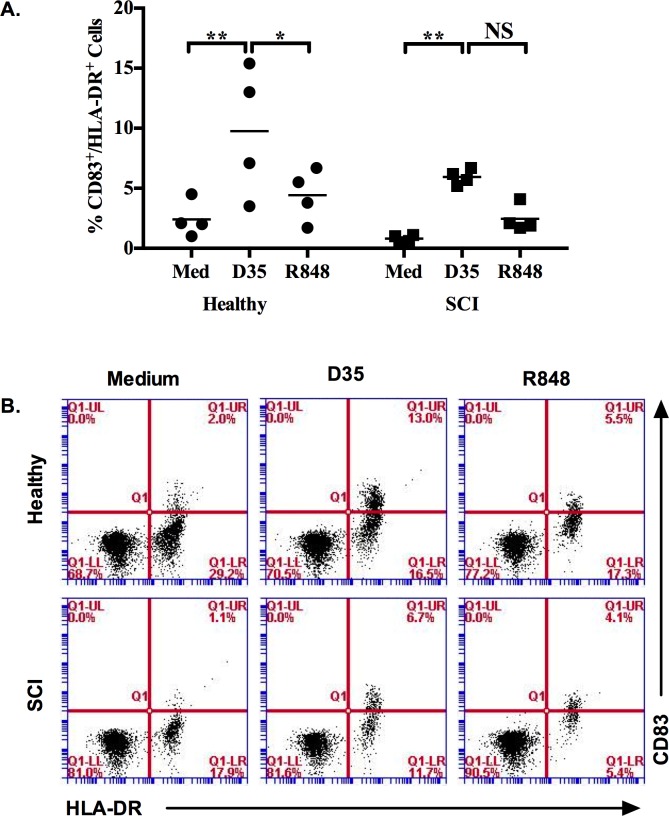
Co-stimulatory molecule and MHC-II expressions altered in response to TLR ligand stimulation of healthy and SCI PBMCs. PBMCs (10^6^/ml) were stimulated with R848 (5μg/ml) or D35 (3μM) for 24h. (A) Average levels of CD83/HLA-DR double positive cells (n = 4), (B) Representative dot plots demonstrating double positive cell percent from PBMCs. During analyses 30000 cells were acquired. Two-way ANOVA with Bonferroni Correction was used to test the significance when compared to medium alone group. (*, p≤0.05; **, p≤0.01).

Off note, the APC function of 4 SCI and 4 healthy PBMCs were analyzed in response to R848 and D35 (Fig 3). Only TLR9 ligand D35 stimulation resulted significantly higher CD83+/HLA-DR+ cells both in healthy and SCI PBMCs. There were less CD83/HLA-DR double positive cells % in response to TLR7 and 9 triggering in SCI compared to healthy PBMCs (Fig 3A and 3B, S3 Fig).

**Fig 4:**
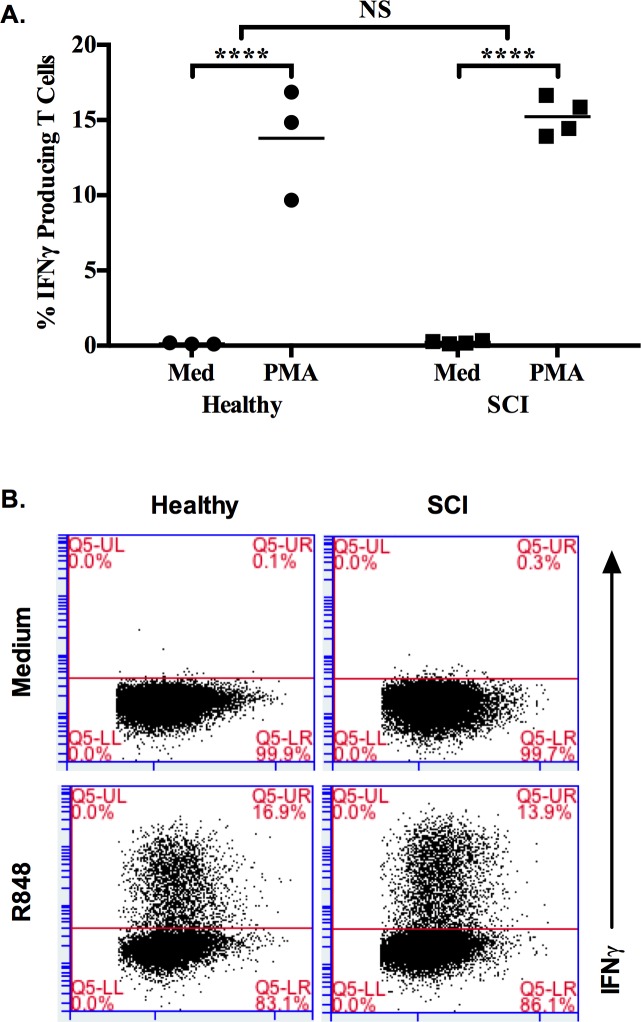
PMA-Ionomycin stimulation induced similar IFNγ producing T cells from healthy and SCI PBMCs. PBMCs (10^6^/ml) were incubated with PMA-Ionomycin for 6h in the presence of Brefeldin A (10μl/ml). (A) % IFNγ producing T cell number by FACS analysis (Healthy = 3, SCI = 4), (B) Representative dot plots showing IFNγ^+^/CD4^+^ T cells. 50000 cells were acquired for the analysis. Mann-Whitney test was used to test significance between healthy and SCI responses. Two-way ANOVA with Bonferroni Correction was used to test the significance when compared to medium alone group. (NS, not significant; ****, p≤0.0001).

Next, function of T cells was evaluated at cellular level by treating PMBCs from 3 healthy and 4 SCI donors with PMA-Ionomycin in the presence of Brefeldin-A. FACS analysis revealed that both healthy and SCI PBMCs had significantly increased number of IFNγ producing T cells (Fig 4A and 4B, S4 Fig). This data demonstrated that dysfunctional immunity is not due to a defect in Th-1 arm of immune activation of SCI patient.

**Fig 5:**
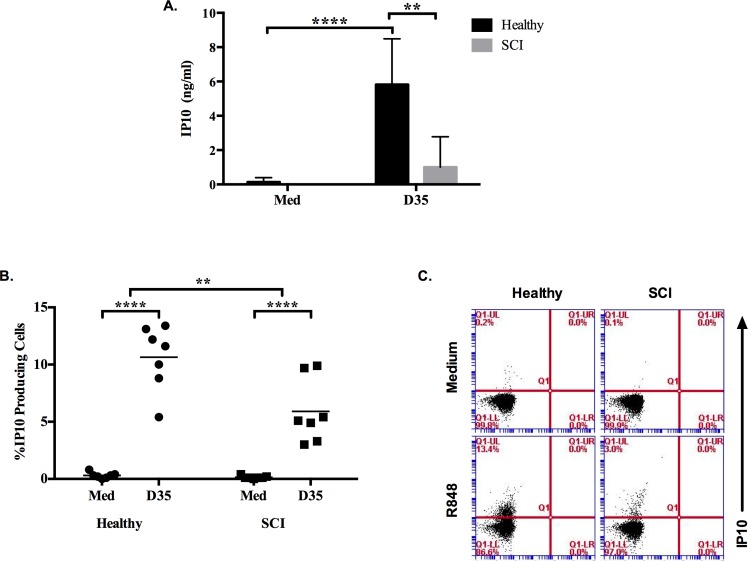
SCI monocytes are defective in IP10 production in response to D35 stimulation. PBMCs (2x10^5^/200μl/well) were incubated with D35 (3μM) for 24h. (A) IP10 secretion levels of healthy (n = 5) and SCI (n = 10) PBMC supernatants. PBMCs (10^6^/ml) were stimulated with D35 (3μM) for 20h, at 12h Brefeldin A (10μl/ml) was added. (B) Percent IP10 producing cells following intracellular cytokine staining of healthy (n = 7) and SCI (n = 7) PBMCs, (C) Representative dot plots showing IP10 producing monocytes. 30000 cells were acquired during the analysis. Mann-Whitney test was used to test significance between healthy and SCI responses to D35. Two-way ANOVA with Bonferroni Correction was used to test the significance when compared to medium alone group. (*, p≤0.05; **, p≤0.01; ****, p≤0.0001).

Moreover, from these PBMCs, we identified that the ability of monocytes to secrete IP10 was significantly impaired in response to D35 treatment. ELISA study suggested that IP10 production by healthy PBMCs but not SCI was significantly increased in response to D35 (Fig 5A). This increased IP10 production was further confirmed at cellular level by FACS analysis. The percentage of CD14^+^/IP10^+^ healthy monocytes significantly increased in response to TLR9 ligand stimulation (Fig 5B and 5C, S5 Fig).

When taken together our results revealed that although chronic SCI patient T cell responses to PMA-Ionomycin stimuli is functional, their monocytes are impaired in in response to both TLR7 and TLR9 ligand stimulations. These defects could contribute to persistent complications due to increased susceptibility to infections.

## Discussion

In this study, we analyzed the status of innate and adaptive immune activation capacities of SCI patient PBMCs. The results revealed that there is a TLR7 and TLR9 mediated innate immune dysfunction in chronic phase of spinal cord injury. We further demonstrated that the diminished T-cell response is not due to the increase in CD4+ regulatory T-cells mediated IL10, but due to impaired Th1-biased IFNγ response. Furthermore, our findings suggested that although there is no significant reduction in response to TLR7 and 9 ligands by B-cells and pDCs, the ability of monocytes to get activated is defective (as evidenced by reduced IP10) in response to the existing innate cytokine milieu.

When animal and human data from the literature is taken into account, immune system dysfunction studies concerning innate and adaptive immune function of SCI patients are ambiguous [4–7, 16]. Furthermore, to our knowledge, there is no study attempted to establish the connection between `TLR’ mediated activation and their contribution to the immune dysfunction. Toll-like receptors are expressed on monocytes/macrophages, dendritic cells, T and B-lymphocytes of immune system cells. Stimulation of TLRs via PAMPS triggers multiple intracellular signaling cascades and establishes an inflammatory cytokine/chemokine milieu and contributes to shape adaptive immune responses [17, 18, 19]. In addition, TLRs are able to recognize endogenous molecules, which are called as danger-associated molecular patterns (DAMPs) [20, 21, 22]. This recognition results with sterile inflammatory processes in various pathological states [23].

In our study, SCI patients and healthy donors presented similar response to TLR7 and TLR9 ligands which implicates that the dysfunction in SCI patients is not due impaired pDC or B cell functions (Fig 1A and 1B). In previous studies regarding innate immune responses of SCI patients, Riegger et al, showed a decrease in ED9^+^ monocytes on early stages of experimental spinal cord injury rat model. But they reported that, on the following days of the injury (day 14), ED9^+^ monocytes recovered to almost control levels. Also, in the same study, HIS 48^+^ granulocyte levels of animals were not different from sham operated controls on day 14 [16]. Riegger et al [24] performed another study in humans and demonstrated that CD14^+^ monocytes decreased on the first week of spinal cord injury but on chronic stages (day 105–136), monocyte number reached a plateau control levels and there is no decrease in CD15^+^ granulocyte number. Here, we demonstrated that although the numbers of monocytes are similar between SCI vs. healthy subjects, their ability to secrete one of the cardinal monocyte-specific IP10 cytokine is impaired in response to R848 and D35 (Fig 2A and Fig 5A and 5B).

Impaired natural killer (NK) cell function is another difference demonstrated in patients with SCI. Cruse et al [25] and Campagnolo et al [4] showed NK cell dysfunction in their studies. In the study of Campagnolo et al [4], the patients were divided into two groups according to level of injury. Although they did not find statistically significant difference between the groups above and below T-6 level, they showed reduction in NK cell numbers and cytotoxicity when compared with healthy group. In present study, reduced IFNγ levels in response to TLR ligand stimulation could support this view, although we did not elaborated on NK-specific interferon production. Further studies are planned to understand this issue.

Here, additionally we checked T cell response rates of both groups via intracellular cytokine staining method. The patient group demonstrated similar increase in T cell response against healthy control group (Fig 1D). This finding was also supported at the cellular level since both SCI patients and healthy donors had almost same level of increase in IFNγ producing T cells in response PMA-Ionomycin (Fig 4A). This finding is in contrast to the data of Campagnolo et al [4]. They showed a significant increase in the percent of T cells and helper cells (CD3^+^, CD4^+^) in SCI patients when compared with healthy controls. Furthermore, T cell responsiveness was emphasized in several other studies [5, 16, 25], however, in these reports, patients were predominantly in acute or sub-acute stages of spinal cord injury limiting accurate comparison with regard to the findings in the present study.

There are some limitations of our study. The sample size of each experiment was different and in some experiments, sample size of patient and healthy subjects was relatively limited. But the unique strengths of the present study rely on the patient inclusion criteria. Here, we intentionally included only complete SCI patients above level of T-6 to exclude the potential impact of the autonomic nerve system on the immune system and included only chronic SCI patients in order to exclude the effects of both infections of the acute and sub-acute stages and the obscuring contributions of medication such as steroids.

## Conclusion

These results might explain the underlying cause for infection susceptibility in the chronic phase of spinal cord injury, especially concerning the innate immune mediated immune activations. More detailed analyses should be planned to explore potential therapeutic targets, thereby helping chronic SCI patients to alleviate susceptibility to infection.

## Supporting information

S1 Fig Plot Illustrating post injury time of SCI patients (n = 17). (TIFF)

S1 Table Detailed information about the antibodies used throughout the study. (A) ELISA and FACS antibodies, (B) Recombinant standard antibodies used in the ELISA studies.
(TIFF)

S2 Fig Representative FACS analysis plots of IFNγ producing T cells. Healthy donor (upper panels) and SCI patient (lower panels) PBMCs were incubated in either medium only or R848. IFNγ signal was detected from CD4^+^ positive T cells.
(TIFF)

S3 Fig Representative FACS analysis plots of CD83^+^ and HLA-DR^+^ cells. (A) Healthy donor and (B) SCI patient PBMCs were stimulated with either D35 or R848.
(EPS)

S4 Fig Representative FACS analysis plots of IFNγ producing T cells. Healthy donor (upper panels) and SCI patient (lower panels) PBMCs were incubated in either medium only or PMA-Ionomycin. IFNγ signal was detected from CD4^+^positive T cells.
(TIFF)

S5 Fig Representative FACS analysis plots of IP10 producing monocytes. Healthy donor (upper panels) and SCI patient (lower panels) PBMCs were incubated in either medium only or D35. IP10 signal was detected from CD14^+^ positive cells.
(TIFF)
