# Supplementary figures and images for: Impaired toll like receptor-7 and 9 induced immune activation in chronic spinal cord injured patients contributes to immune dysfunction
Source: PLoS One. 2017 Feb 7;12(2):e0171003. doi: 10.1371/journal.pone.0171003 (PMC5295667; doi:10.1371/journal.pone.0171003)

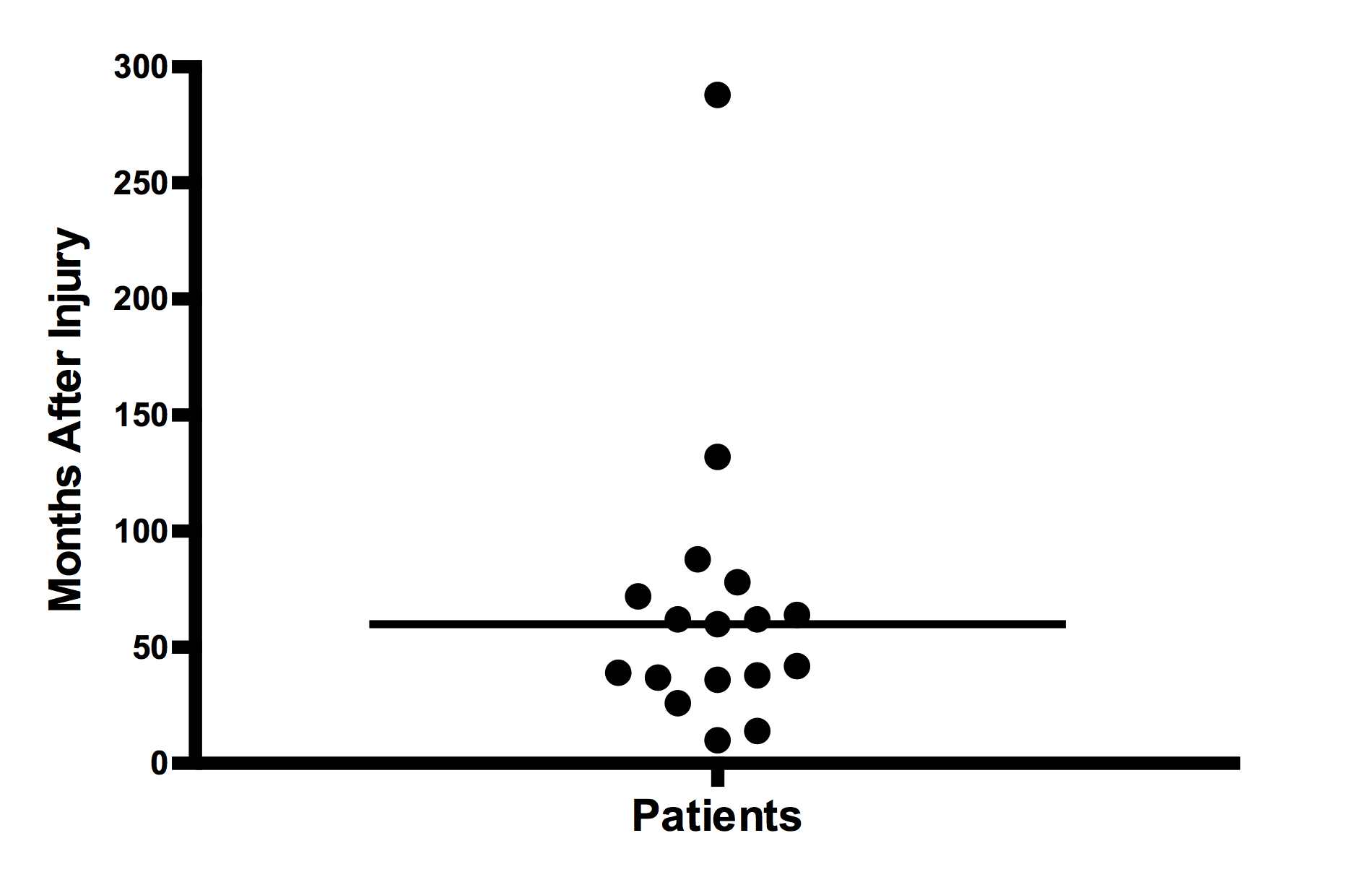

Supplement: S1 Fig — (TIFF) [file pone.0171003.s001.tiff]

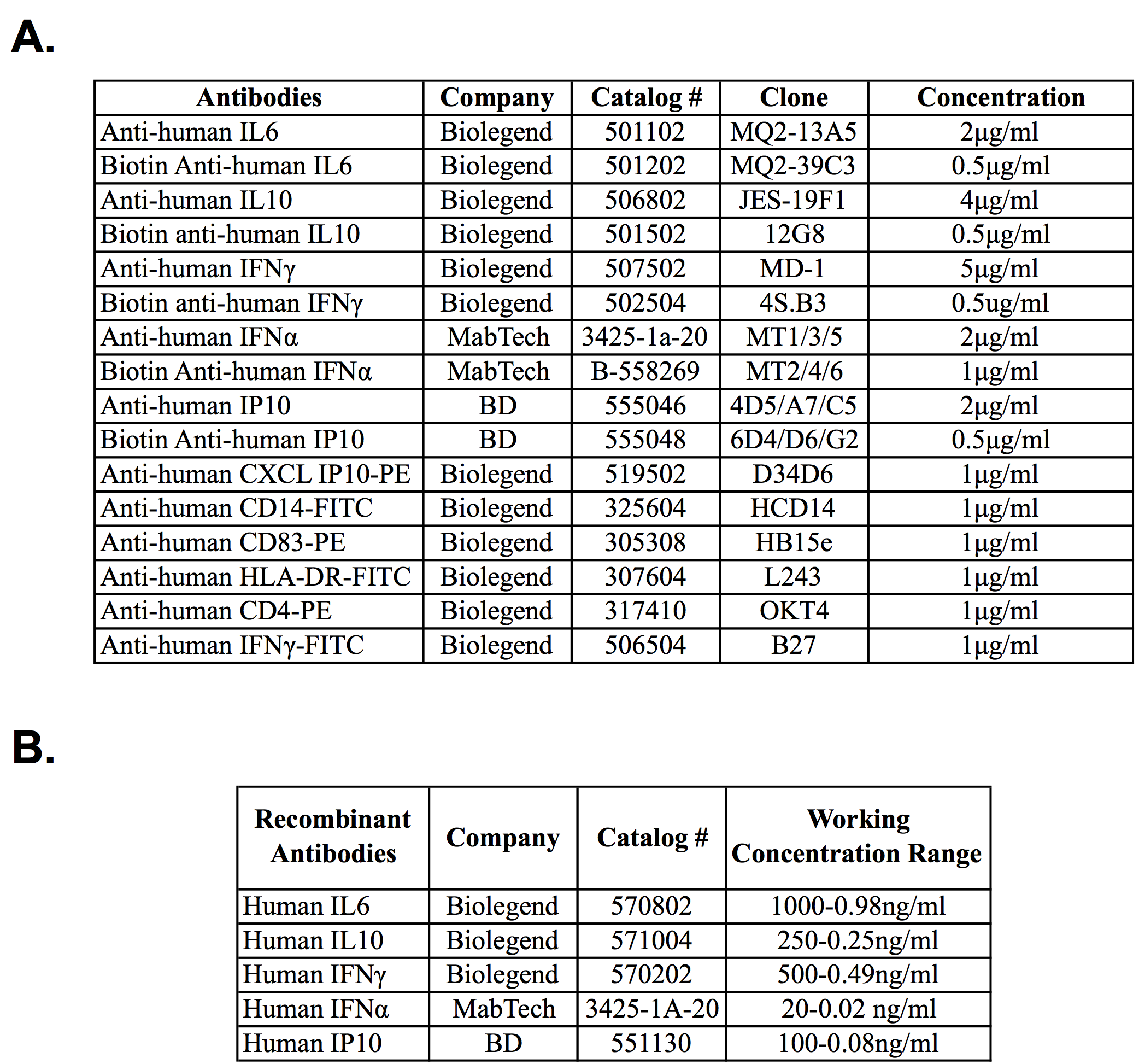

Supplement: S1 Table — (A) ELISA and FACS antibodies, (B) Recombinant standard antibodies used in the ELISA studies. (TIFF) [file pone.0171003.s002.tiff]

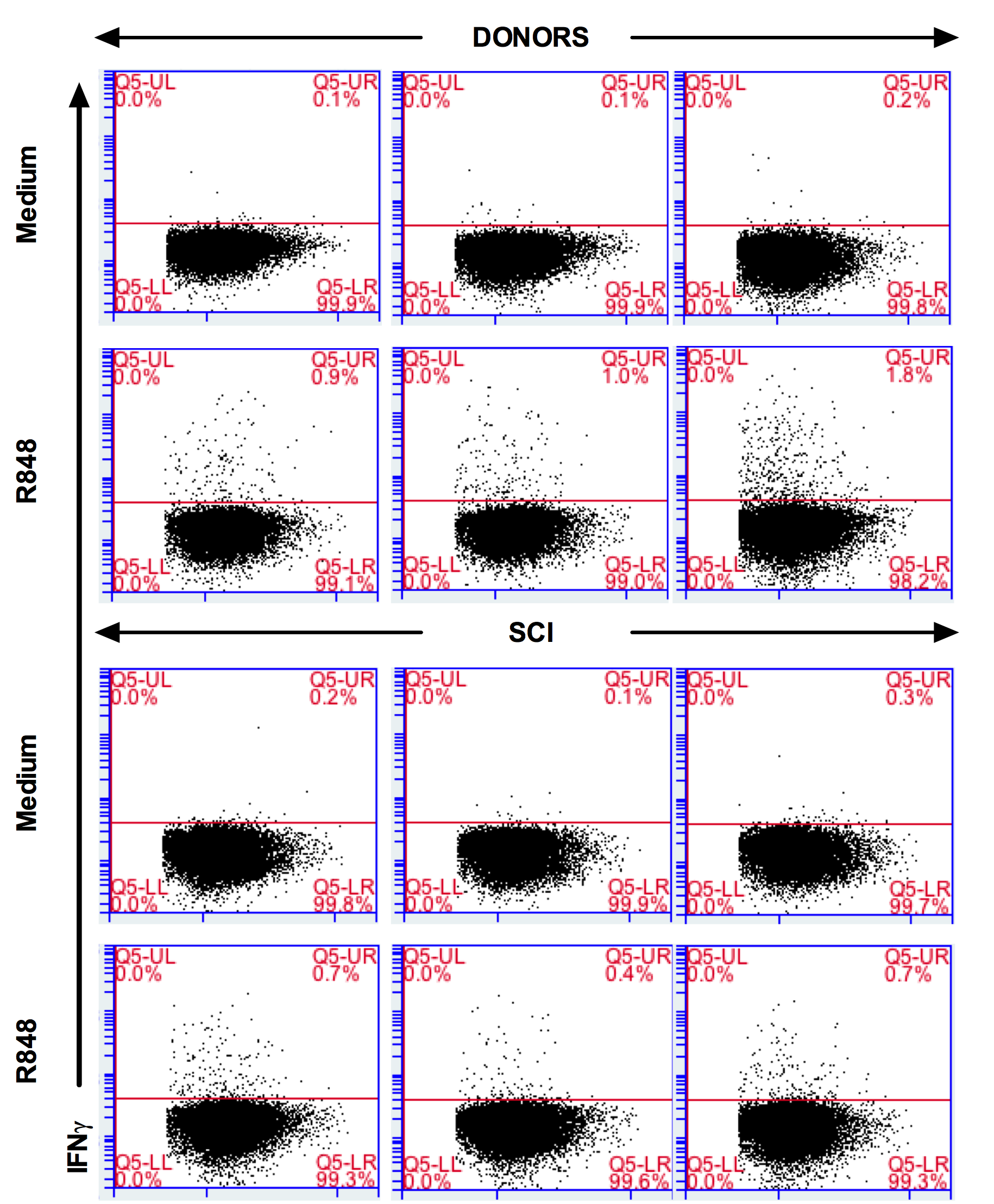

Supplement: S2 Fig — Healthy donor (upper panels) and SCI patient (lower panels) PBMCs were incubated in either medium only or R848. IFNγ signal was detected from CD4+ positive T cells. (TIFF) [file pone.0171003.s003.tiff]

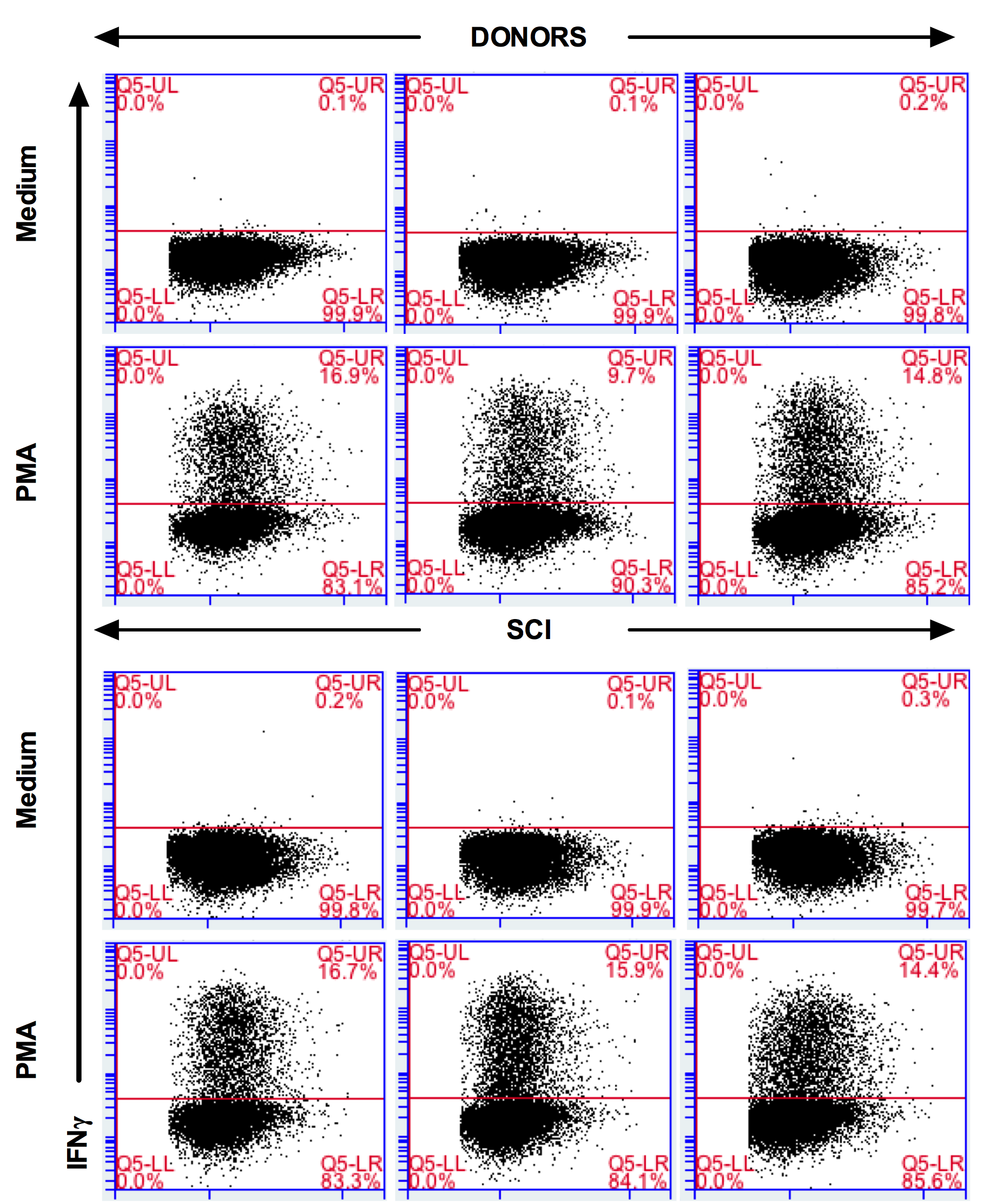

Supplement: S4 Fig — Healthy donor (upper panels) and SCI patient (lower panels) PBMCs were incubated in either medium only or PMA-Ionomycin. IFNγ signal was detected from CD4+positive T cells. (TIFF) [file pone.0171003.s005.tiff]

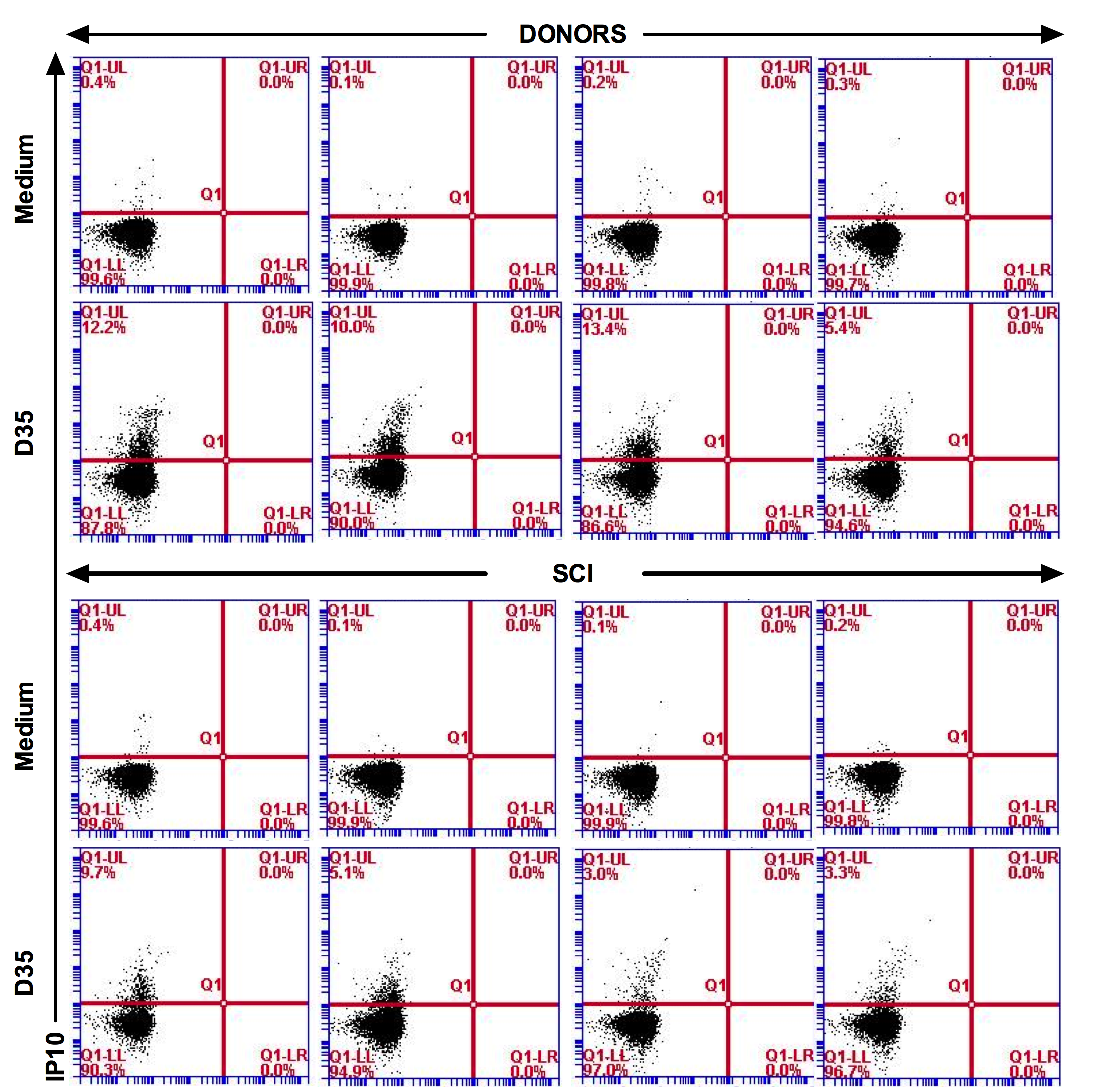

Supplement: S5 Fig — Healthy donor (upper panels) and SCI patient (lower panels) PBMCs were incubated in either medium only or D35. IP10 signal was detected from CD14+ positive cells. (TIFF) [file pone.0171003.s006.tiff]
